# First-trimester atherogenic index of plasma and triglyceride–glucose indices in pregestational diabetes mellitus: associations with adverse pregnancy outcomes

**DOI:** 10.1186/s12884-025-08491-2

**Published:** 2025-11-22

**Authors:** Serenat Eris Yalcin, Nuray Nerez, Yakup Yalcin, Bilge Demir Cetinkaya

**Affiliations:** 1 Department of Obstetrics and Gynecology, Faculty of Medicine, Uludağ University, Bursa, Turkey; 2 Department of Obstetrics and Gynecology, Bursa City Hospital, Bursa, Turkey

**Keywords:** Pregestational diabetes, Atherogenic index of plasma, Triglyceride–glucose index, Pregnancy outcomes, First trimester, Neonatal compromise

## Abstract

**Background:**

Pregestational diabetes mellitus (PREGDM) confers a high risk of maternal and perinatal morbidity. Identifying early, reliable biomarkers is critical for risk stratification. The atherogenic index of plasma (AIP) and triglyceride–glucose (TyG) index are inexpensive metabolic markers reflecting dyslipidemia and insulin resistance, but their role in PREGDM remains underexplored.

**Methods:**

In this retrospective cohort study, medical records of 628 women (314 with PREGDM, 314 controls) delivering at a tertiary referral center were reviewed. First-trimester fasting blood samples were analyzed to calculate AIP [log₁₀(TG/HDL-C)] and TyG [ln(TG×glucose/2)]. Maternal and perinatal outcomes assessed included preeclampsia, preterm birth, macrosomia, fetal growth restriction (FGR), NICU admission, Apgar < 7 at 1 and 5 min, and a composite adverse perinatal outcome (CAPO). Logistic regression adjusted for age, BMI, parity, smoking, HbA1c, and fasting glucose; predictive ability was evaluated by ROC analysis.

**Results:**

Compared with controls, women with PREGDM had significantly higher AIP (0.171 ± 0.207 vs. 0.148 ± 0.029, *p* < 0.001) and TyG (9.33 ± 0.65 vs. 8.90 ± 0.10, *p* < 0.001). PREGDM was associated with higher rates of preeclampsia (13.1% vs. 4.5%), preterm birth (21.7% vs. 10.8%), macrosomia (13.4% vs. 5.7%), FGR (8.3% vs. 3.8%), NICU admission (23.6% vs. 8.9%), and CAPO (29.3% vs. 9.9%) (all *p* < 0.01). In multivariate models, TyG independently predicted preterm birth (OR 1.62, 95% CI 1.28–2.04), macrosomia (OR 1.48, 95% CI 1.10–1.99), CAPO (OR 1.78, 95% CI 1.35–2.33), and low Apgar at 1 min (OR 1.84, 95% CI 1.40–2.42). AIP was inversely associated with FGR (OR 0.68, 95% CI 0.51–0.92). ROC analysis showed high accuracy of TyG for predicting low Apgar at 1 min (AUC 0.88) and NICU admission (AUC 0.76).

**Conclusions:**

Both AIP and TyG are elevated in PREGDM compared with controls. TyG is a robust predictor of preterm birth, macrosomia, neonatal compromise, and CAPO, whereas AIP provides complementary insight into vascular dysfunction and FGR in PREGDM group. These indices, easily derived from routine first-trimester tests, may offer practical tools for early obstetric risk stratification.

**Supplementary Information:**

The online version contains supplementary material available at 10.1186/s12884-025-08491-2.

## Introduction

Diabetes mellitus is a growing global health concern and represents one of the most common medical disorders complicating pregnancy. Pregestational diabetes mellitus (PREGDM), which includes both type 1 and type 2 diabetes, confers a substantially increased risk of maternal and perinatal morbidity compared with the general obstetric population. Women with PREGDM are more likely to experience preeclampsia, preterm delivery, macrosomia, fetal growth restriction (FGR), stillbirth, neonatal hypoglycemia, and admission of their infants to neonatal intensive care units (NICU) [1, 2]. Despite advances in obstetric and neonatal care, identifying reliable predictors of these complications remains an important unmet need.

The mechanisms driving adverse outcomes in diabetic pregnancies are complex and multifactorial, with dyslipidemia and insulin resistance playing pivotal roles. Insulin resistance not only underpins the metabolic dysregulation of type 2 diabetes and gestational diabetes mellitus (GDM) but also contributes to endothelial dysfunction and impaired placental vascular adaptation, which are central to the pathogenesis of preeclampsia and fetal growth restriction [3, 4]. Traditional lipid parameters, such as total cholesterol, triglycerides, and low- or high-density lipoproteins, are insufficient to capture this complexity and provide limited predictive value. This has led to the development of non-traditional indices that combine multiple metabolic components to better reflect cardiometabolic risk [4–7].

The Atherogenic Index of Plasma (AIP), calculated as the logarithm of the triglyceride-to-HDL cholesterol ratio, integrates the balance between atherogenic and protective lipoproteins. AIP has been linked to cardiovascular disease, metabolic syndrome, and insulin resistance, and more recently has been associated with pregnancy complications such as preeclampsia, abnormal fetal growth, and other adverse perinatal outcomes [8–16]. Similarly, the Triglyceride–Glucose (TyG) index, derived from fasting triglyceride and glucose levels, has emerged as a simple and cost-effective surrogate marker of insulin resistance. It has shown strong correlations with the gold-standard euglycemic clamp and has been validated in the prediction of type 2 diabetes, cardiovascular disease, and metabolic syndrome [8, 17]. Importantly, TyG levels measured in early pregnancy have been shown to predict subsequent development of GDM and preeclampsia [18–21].

Recent research has also expanded these indices to composite forms, such as the TyG-BMI, which combines TyG with body mass index and has demonstrated improved predictive performance for GDM [6]. These findings highlight the potential utility of non-traditional metabolic indices in obstetric risk stratification. However, most available evidence is derived from women with GDM, while data regarding their role in pregnancies complicated by pregestational diabetes are extremely limited. This gap is particularly relevant, as women with PREGDM carry a higher baseline metabolic burden and more profound insulin resistance than those who develop hyperglycemia only during pregnancy, potentially amplifying the prognostic value of indices such as AIP and TyG.

In addition to short-term obstetric risks, there is increasing recognition that pregnancy acts as a “stress test” for maternal cardiovascular health. Placental pathology studies have demonstrated that maternal vascular malperfusion lesions, often associated with dyslipidemia and impaired endothelial function, not only predict preeclampsia and fetal growth restriction but also herald long-term vascular dysfunction in mothers [5]. Thus, investigating metabolic indices that link lipid and glucose metabolism with pregnancy outcomes may provide insights not only into perinatal prognosis but also into women’s future cardiovascular risk.

This study had two complementary objectives: (1) to compare first-trimester AIP and TyG levels between women with PREGDM and healthy controls, and (2) to investigate, within the PREGDM group, whether these indices are associated with adverse pregnancy outcomes. The first objective follows a case–control design, whereas the second represents a cohort analysis restricted to PREGDM participants.

## Materials and methods

### Study design and population

**Fig. 1:**
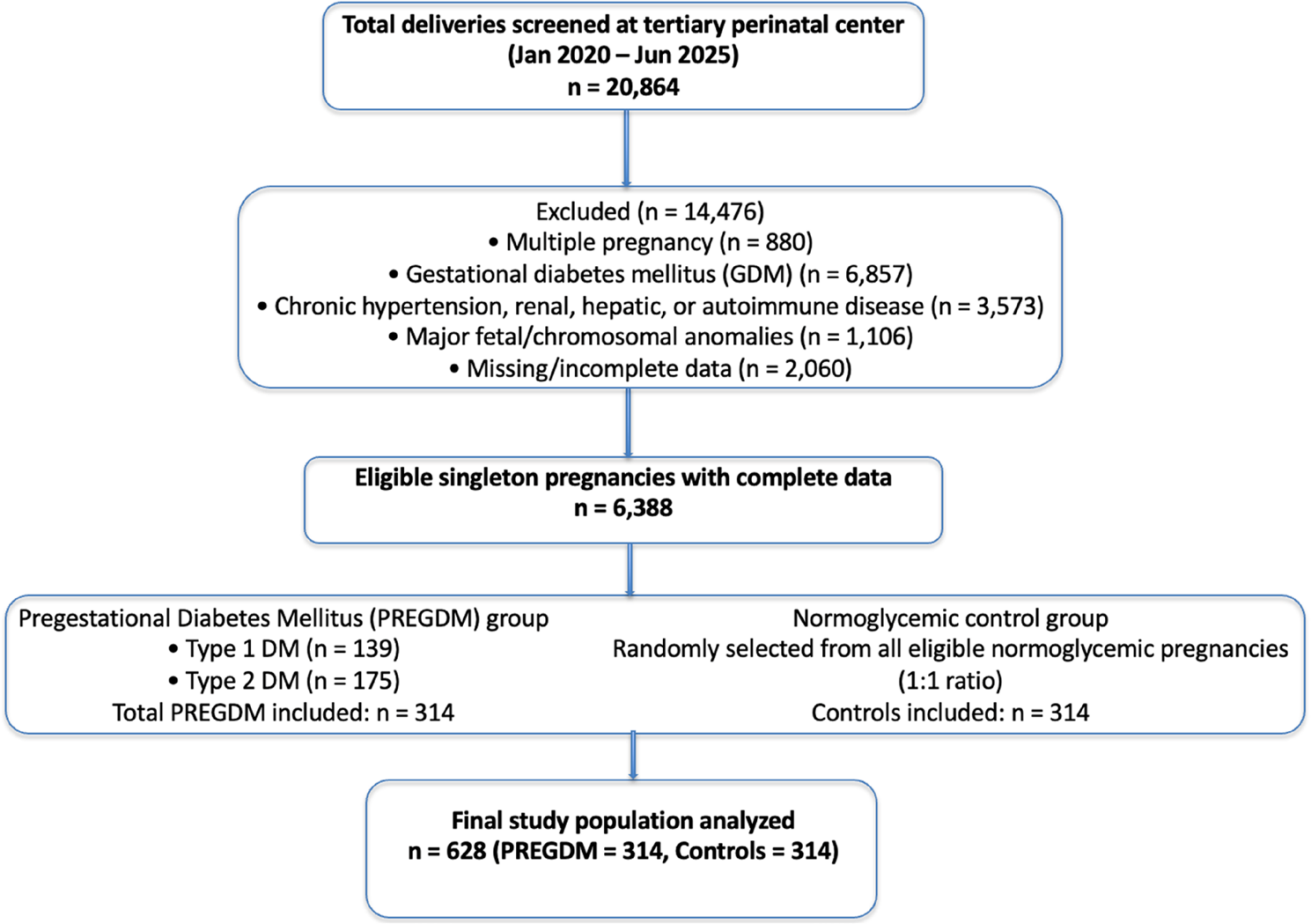
Flowchart of study population selection

This retrospective cohort study was conducted at a tertiary referral center for high-risk pregnancies. Medical records of women who delivered between January 2020 and June 2025 were reviewed. A total of 20,864 deliveries were screened during this period, of which 628 participants met the inclusion criteria and were included in the final analysis; 314 women with PREGDM and 314 healthy controls. All women were followed from the first trimester until delivery. Control participants were randomly selected from all eligible normoglycemic pregnancies delivered during the same study period to achieve a 1:1 ratio with the PREGDM group. No individual matching was performed; instead, regression analyses were adjusted for relevant confounders including maternal age, BMI, parity, smoking status, and HbA1c. The process of screening, exclusion, and final inclusion of participants is summarized in Fig. 1.

Two analytical approaches were adopted: a case–control comparison of metabolic indices between PREGDM and controls, and a cohort-type analysis assessing associations of AIP and TyG with outcomes within PREGDM.

The PREGDM group consisted of women diagnosed with type 1 or type 2 diabetes mellitus before conception, confirmed through medical records or pre-pregnancy laboratory documentation, in accordance with the American Diabetes Association (ADA) diagnostic criteria; type 1 diabetes mellitus (T1DM) is defined by autoimmune β-cell destruction leading to absolute insulin deficiency, typically early onset, low/undetectable C-peptide (< 200 pmol/L), and positivity for pancreatic autoantibodies. Type 2 diabetes mellitus (T2DM) is defined by standard diagnostic thresholds: HbA1c ≥ 6.5%, fasting plasma glucose ≥ 126 mg/dL, 2-h OGTT ≥ 200 mg/dL, or random plasma glucose ≥ 200 mg/dL with classic hyperglycemic symptoms [22].

Controls were selected from women who delivered in the same period, had normal glucose screening results, and had no pregestational or gestational diabetes. Participants with major systemic illnesses were excluded. No additional matching was performed; therefore, differences in baseline characteristics such as maternal age were evaluated in the statistical analysis. The study was approved by the Ethics Committee of Bursa City Hospital (Approval No: 2025-8/12) and conducted in accordance with the Declaration of Helsinki.

### Inclusion and exclusion criteria

#### Inclusion criteria (PREGDM group)

1. Singleton pregnancy.
2. Documented diagnosis of type 1 or type 2 diabetes before conception.
3. Availability of complete clinical and laboratory data.

#### Exclusion criteria (both groups)

- Multiple pregnancy
- GDM
- Major congenital anomalies or chromosomal abnormalities
- Hyperthyroidism, systemic lupus erythematosus, rheumatoid arthritis, cardiovascular disease, chronic hypertension, kidney or liver disease
- Incomplete or missing data

GDM was excluded from both groups to ensure that all diabetic participants had pregestational rather than pregnancy-onset diabetes.

### Data collection

Maternal demographic and obstetric characteristics (such as age, BMI, gravida, parity, smoking status, history of hypertension) and laboratory findings (fasting glucose, HbA1c, lipid profile including triglycerides, total cholesterol, HDL-C, LDL-C) were extracted from electronic medical records. Blood samples were collected between 8 and 13 + 6 weeks of gestation as part of routine first-trimester screening. Urine dipstick test results from the same visit were also reviewed to assess the presence of proteinuria, which was defined as ≥ 1 + on dipstick analysis. Blood pressure measurements recorded at the first antenatal visit were retrieved from electronic medical records. The systolic and diastolic values obtained during the same visit as laboratory sampling were used for analysis. Smoking status was self-reported at the first antenatal visit.

Two indices were calculated:

- Atherogenic Index of Plasma (AIP): log₁₀ [TG/HDL-C] (mmol/L).
- Triglyceride–Glucose (TyG) index: ln [fasting triglyceride (mg/dL) × fasting glucose (mg/dL)/2]

TyG and AIP were selected a priori based on their validated use in metabolic and cardiovascular risk prediction, and their previously demonstrated correlations with insulin resistance and endothelial dysfunction in both diabetic and obstetric populations.

Data regarding specific insulin regimens or use of lipid-lowering medications were unavailable and could not be included as covariates.

### Pregnancy outcomes

The maternal and perinatal outcomes assessed were:

- Preeclampsia: defined according to ISSHP criteria (new-onset hypertension with proteinuria or end-organ dysfunction after 20 weeks) [23].
- Preterm birth: delivery before 37 weeks
- Macrosomia: birthweight ≥ 4000 g
- Fetal growth restriction (FGR): FGR was defined based on the Delphi consensus definition, which incorporates fetal biometric parameters and Doppler findings to distinguish pathological growth restriction from constitutionally small fetuses [24].
- Low Apgar score: <7 at 1 and/or 5 minutes
- NICU admission
- Composite Adverse Perinatal Outcome (CAPO): defined as the occurrence of any of the above outcomes

### Statistical analysis

Continuous variables were expressed as mean ± standard deviation or median (IQR), and categorical variables as number (percentage). Normality was assessed using the Shapiro–Wilk test. Comparisons between PREGDM and controls were performed using the Mann–Whitney U **test** for continuous variables and the chi-square or Fisher’s exact test for categorical variables. Within the PREGDM group, associations between AIP, TyG, and adverse outcomes were explored using Mann–Whitney U tests.

Multivariate logistic regression analyses were conducted to determine the independent predictive value of AIP and TyG, adjusting for maternal age, BMI, parity, smoking, HbA1c, and fasting glucose. Odds ratios (OR) with 95% confidence intervals (CI) were reported. The discriminative ability of AIP and TyG for predicting outcomes was evaluated by **receiver** operating characteristic (ROC) curves and area under the curve (AUC) analysis. A two-tailed p-value < 0.05 was considered statistically significant.

An a priori power analysis (GPower, version 3.1.9.7, University of Düsseldorf, Germany) was performed for the primary outcome (between-group differences in AIP and TyG as continuous variables). Assuming a two-sided α = 0.05 and 80% power, the study would require 286 participants per group to detect a standardized mean difference of Cohen’s d = 0.25–0.30 (range reported in prior lipid-index literature in pregnancy). In addition, a post hoc power calculation confirmed that the achieved sample (*n* = 314 per group) provided ≥ 80% power to detect moderate effect sizes (Cohen’s d ≥ 0.25) for between-group comparisons and odds ratios ≥ 1.5 for within-group associations.

For the secondary outcome (associations of AIP and TyG with adverse pregnancy outcomes within the PREGDM group), multivariable logistic regression models were planned with adjustment for prespecified covariates (maternal age, BMI, parity, smoking, HbA1c, and fasting glucose). With *n* = 314 PREGDM and outcome incidences typically observed in PREGDM pregnancies (e.g., ≈ 15–25% for preeclampsia/NICU admission), the sample affords ≥ 10 events per variable (EPV) for models including 5–6 predictors, thereby meeting accepted stability thresholds. Under these event rates, the study has ≈ 80% power to detect odds ratios (OR) on the order of 1.45–1.60 per 1-SD increase in AIP or TyG. Discriminative performance was evaluated by ROC analysis; with the available sample and event fractions in this range, the study has adequate power to detect AUC ≥ 0.60–0.62 vs. 0.50 (α = 0.05, two-sided).

To enhance transparency, effect sizes are reported as standardized mean differences (primary analysis) and adjusted OR per 1-SD increase (secondary analysis), with 95% confidence intervals. All power assumptions were specified a priori; no data-driven recalibration of α or multiplicity adjustment was applied, as the two prespecified indices (AIP, TyG) and outcomes were analyzed within a predefined hierarchy (primary between-group comparisons; secondary within-PREGDM prognostic analyses).

## Results

### Study population

A total of 628 pregnant women were included in the analysis, comprising 314 PREGDM cases and 314 matched controls. The PREGDM group comprised 139 (44.3%) women with T1DM and 175 (55.7%) with T2DM.

### Maternal demographic and metabolic characteristics

Baseline characteristics are summarized in Table 1. Maternal age, BMI, gravida, and parity were significantly higher in the PREGDM group, whereas smoking rates were similar between groups. A history of abortion was also more frequent in the PREGDM group (44.3% vs. 21.3%, *p* < 0.001).

**Table 1:** Maternal demographic, clinical, and laboratory characteristics in women with PREGDM and controls

Variable	PREGDM (*n* = 314)	Control (*n* = 314)	*p*-value
Age (years)	32.37 ± 5.74	29.74 ± 3.13	< 0.0001
BMI (kg/m²)	30.53 ± 6.32	25.83 ± 1.95	< 0.0001
Smoking, n (%)	33 (10.5%)	36 (11.5%)	0.799
Gravida	3 (2–4)	2 (1–3)	< 0.001
Parity	2 (1–3)	1 (0–2)	< 0.001
History of abortion, n (%)	139 (44.3%)	67 (21.3%)	< 0.001
Gestational age at delivery (weeks)	36.7 ± 2.7	38.6 ± 1.0	< 0.001
Birthweight (g)	3188 ± 750	3429 ± 334	< 0.001
HOMA-IR	5.92 ± 2.96	2.58 ± 0.22	< 0.0001
HbA1c (%)	6.59 ± 1.37	4.93 ± 0.37	< 0.0001
Insulin (µU/mL)	18.52 ± 2.91	12.64 ± 0.90	< 0.0001
Atherogenic index (AIP)	0.171 ± 0.207	0.148 ± 0.029	0.0001
TyG index	9.33 ± 0.65	8.90 ± 0.10	< 0.0001
Systolic BP (mmHg)	116.4 ± 10.3	113.6 ± 9.8	0.0005
Diastolic BP (mmHg)	81.9 ± 8.1	80.2 ± 7.5	0.0074
FBG (mg/dL)	128.3 ± 53.1	82.7 ± 5.3	< 0.0001
AST (IU/L)	16.01 ± 6.56	17.03 ± 6.69	0.022
ALT (IU/L)	12.79 ± 6.55	16.51 ± 7.14	< 0.0001
WBC (/mm³)	11710.9 ± 10544.4	7598.4 ± 1640.8	< 0.0001
Neutrophils (/mm³)	7155.1 ± 3198.1	4498.9 ± 928.1	< 0.0001
Lymphocytes (/mm³)	1985.4 ± 1157.5	1843.5 ± 674.2	0.618
Monocytes (/mm³)	657.4 ± 248.0	528.7 ± 140.7	< 0.0001
Platelets (/mm³)	244116.4 ± 89056.2	215628.5 ± 40468.9	0.0001
Triglycerides (mg/dL)	212.5 ± 61.4	142.8 ± 38.6	< 0.0001
LDL cholesterol (mg/dL)	123.6 ± 38.1	104.7 ± 29.5	< 0.0001
HDL cholesterol (mg/dL)	46.8 ± 8.2	55.6 ± 9.3	< 0.0001
Total cholesterol (mg/dL)	218.4 ± 41.7	196.2 ± 35.5	< 0.0001
Urine protein (dipstick positive), n (%)	61 (19.4%)	21 (6.7%)	< 0.001

Continuous variables are presented as mean ± standard deviation or median (interquartile range); categorical variables are presented as n (%). Between-group comparisons were performed using the Mann–Whitney U test for continuous variables and the chi-square or Fisher’s exact test for categorical variables. *P* < 0.05 was considered statistically significant

HOMA-IR was calculated as [fasting insulin × fasting glucose] / 405. AIP was defined as log₁₀(TG/HDL-C) (mmol/L), and TyG index as ln [TG (mg/dL) × fasting glucose (mg/dL) / 2]

*PREGDM* pregestational diabetes mellitus, *BMI* body mass index, *HbA1c* glycated hemoglobin, *HOMA-IR* homeostatic model assessment of insulin resistance, *AIP* atherogenic index of plasma, *TyG* triglyceride–glucose index, *FBG* fasting blood glucose, *AST* aspartate aminotransferase, *ALT* alanine aminotransferase, *WBC* white blood cell count, *LDL* low-density lipoprotein cholesterol, *HDL* high-density lipoprotein cholesterol, *NICU* neonatal intensive care unit

Additionally, both systolic and diastolic blood pressures were modestly but significantly higher in the PREGDM group compared with controls (116.4 ± 10.3 vs. 113.6 ± 9.8 mmHg, *p* = 0.0005; and 81.9 ± 8.1 vs. 80.2 ± 7.5 mmHg, *p* = 0.007, respectively), although the values remained within the normotensive range.

Delivery outcomes differed significantly between groups. Mean gestational age at delivery was lower in women with PREGDM compared with controls (36.7 ± 2.7 vs. 38.6 ± 1.0 weeks, *p* < 0.001). Similarly, mean birthweight was significantly lower in the PREGDM group (3188 ± 750 g vs. 3429 ± 334 g, *p* < 0.001). Cesarean delivery was substantially more common in PREGDM pregnancies (74.2% vs. 43.0%, *p* < 0.001).

Among cesarean indications, previous cesarean section was the leading reason in both groups but was more common in PREGDM (33.8% vs. 11.8%). In addition, macrosomia-related cesarean was nearly twice as frequent in PREGDM women (9.2% vs. 4.1%). Cephalopelvic disproportion (13.4% vs. 12.7%) and fetal distress (8.9% vs. 8.6%) were comparable between groups, while presentation anomalies were slightly more common in PREGDM (7.6% vs. 4.5%).

**Fig. 2:**
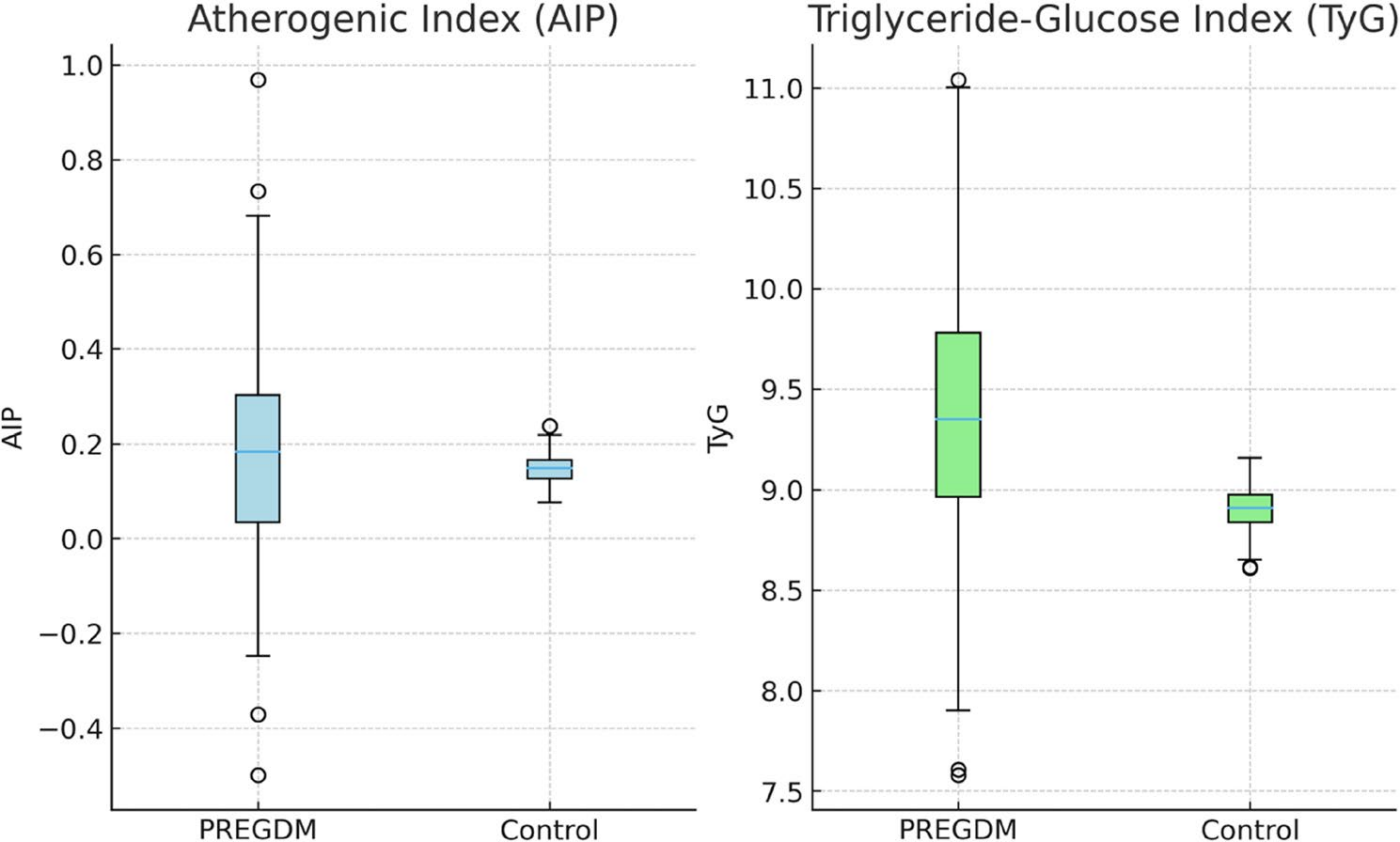
Comparison of AIP and TyG between groups

Regarding laboratory parameters, PREGDM women exhibited higher HbA1c, fasting glucose, fasting insulin, and HOMA-IR values compared with controls (all *p* < 0.001). Their lipid profile was also more atherogenic, with significantly higher triglycerides, total cholesterol, and LDL, and lower HDL levels. Consequently, both the Atherogenic Index of Plasma (AIP) and the Triglyceride-Glucose (TyG) index were markedly elevated in the PREGDM group compared with controls (both *p* < 0.001) (Fig. 2). Proteinuria (dipstick positive) was significantly more frequent in women with PREGDM than in controls (19.4% vs. 6.7%, *p* < 0.001).

### Adverse pregnancy outcomes

Adverse maternal and neonatal outcomes are summarized in Table 2. Women with PREGDM experienced significantly higher rates of preeclampsia (13.1% vs. 4.5%), preterm birth < 37 weeks (21.7% vs. 10.8%), macrosomia (13.4% vs. 5.7%), FGR (8.3% vs. 3.8%), NICU admission (23.6% vs. 8.9%), and CAPO (29.3% vs. 9.9%) (all *p* < 0.01).

**Table 2:** Pregnancy and neonatal outcomes in women with PREGDM and controls

Outcome	PREGDM (*n* = 314)	Control (*n* = 314)	*p*-value
Preterm birth (< 37 weeks)	68 (21.7%)	34 (10.8%)	0.0002
Macrosomia (> 4000 g)	42 (13.4%)	18 (5.7%)	0.0015
FGR	26 (8.3%)	12 (3.8%)	0.018
NICU admission	74 (23.6%)	28 (8.9%)	< 0.0001
Apgar score < 7 (1 min)	59 (18.8%)	16 (5.1%)	< 0.0001
Apgar score < 7 (5 min)	34 (10.8%)	10 (3.2%)	0.001
Composite adverse perinatal outcome (CAPO)	92 (29.3%)	31 (9.9%)	< 0.0001
Preeclampsia	41 (13.1%)	14 (4.5%)	0.0003
Cesarean delivery	233 (74.2%)	135 (43.0%)	<0.001
Stillbirth	6 (1.9%)	2 (0.6%)	0.16

Continuous variables are presented as mean ± standard deviation; categorical variables are presented as n (%)

Between-group comparisons were performed using the chi-square test or Fisher’s exact test as appropriate

*P* < 0.05 was considered statistically significant

*PREGDM* pregestational diabetes mellitus, *NICU* neonatal intensive care unit, *FGR* fetal growth restriction, *CAPO* composite adverse perinatal outcome, *CS* cesarean section

CAPO was defined as the occurrence of any of the following outcomes: preeclampsia, preterm birth, macrosomia, FGR, NICU admission, or low Apgar score

In addition, Apgar < 7 at 1 and 5 min was more frequent in the diabetic group (18.8% vs. 5.1% and 10.8% vs. 3.2%, respectively).

### Univariate analysis within PREGDM

Univariate logistic regression results are displayed in Table 3. Within the PREGDM cohort, higher TyG values were significantly associated with an increased risk of preterm birth (OR 1.45, 95% CI 1.18–1.79), macrosomia (OR 1.38, 95% CI 1.07–1.77), NICU admission (OR 1.52, 95% CI 1.23–1.89), CAPO (OR 1.78, 95% CI 1.35–2.33), and low Apgar scores at 1 and 5 min (all *p* < 0.01).

**Table 3:** Univariate logistic regression of AIP and TyG for adverse pregnancy outcomes in women with PREGDM

Outcome	Index	OR (95% CI)	*p*-value
Preterm birth (< 37 wk)	TyG	1.45 (1.18–1.79)	< 0.001
	AIP	1.12 (0.88–1.43)	0.34
Macrosomia (> 4000 g)	TyG	1.38 (1.07–1.77)	0.012
	AIP	1.09 (0.84–1.41)	0.49
FGR	TyG	0.96 (0.72–1.28)	0.78
	AIP	0.68 (0.51–0.92)	0.013
NICU admission	TyG	1.52 (1.23–1.89)	< 0.001
	AIP	1.15 (0.92–1.46)	0.21
Apgar < 7 (1 min)	TyG	1.84 (1.40–2.42)	< 0.001
	AIP	1.42 (1.08–1.88)	0.012
Apgar < 7 (5 min)	TyG	1.59 (1.16–2.17)	0.004
	AIP	1.28 (0.94–1.74)	0.11
Preeclampsia	TyG	1.21 (0.95–1.54)	0.12
	AIP	1.46 (1.08–1.99)	0.013
CAPO	TyG	1.78 (1.35–2.33)	< 0.001
	AIP	1.56 (1.22–2.00)	< 0.001

Values are presented as odds ratios (OR) with 95% confidence intervals (CI). Logistic regression analyses were performed separately for each outcome and biomarker. *P*-values < 0.05 were considered statistically significant

*AIP* atherogenic index of plasma, *TyG* triglyceride–glucose index, *FGR* fetal growth restriction, *NICU* neonatal intensive care unit, *CAPO* composite adverse perinatal outcome, *OR* odds ratio, *CI* confidence interval

In contrast, AIP was positively associated with low Apgar at 1 min (OR 1.42, 95% CI 1.08–1.88) and preeclampsia (OR 1.46, 95% CI 1.08–1.99), while showing an inverse association with FGR (OR 0.68, 95% CI 0.51–0.92).

### Multivariate logistic regression

After adjustment for maternal age, BMI, parity, smoking, HbA1c, and fasting glucose (Table 4), TyG remained an independent predictor of preterm birth (OR 1.62, 95% CI 1.28–2.04, *p* < 0.001), macrosomia (OR 1.48, 95% CI 1.10–1.99, *p* = 0.008), CAPO (OR 1.78, 95% CI 1.35–2.33, *p* < 0.001), and low Apgar at 1 min (OR 1.84, 95% CI 1.40–2.42, *p* < 0.001).

**Table 4:** Multivariate regression and ROC analysis of AIP and TyG for adverse pregnancy outcomes in women with PREGDM

Outcome	Index	OR (95% CI)	*p*-value	AUC (95% CI)	Cut-off	Sensitivity (%)	Specificity (%)
Preterm birth	TyG	1.62 (1.28–2.04)	**< 0.001**	0.74 (0.68–0.80)	9.15	71	69
Macrosomia	TyG	1.48 (1.10–1.99)	**0.008**	0.70 (0.63–0.77)	9.20	68	65
FGR	AIP	0.68 (0.51–0.92)	**0.013**	0.68 (0.61–0.75)	0.21	66	64
NICU admission	TyG	1.71 (1.32–2.23)	**< 0.001**	0.76 (0.70–0.82)	9.18	73	70
Apgar < 7 (1 min)	TyG	1.84 (1.40–2.42)	**< 0.001**	0.88 (0.83–0.93)	9.12	82	77
Apgar < 7 (5 min)	TyG	1.59 (1.16–2.17)	**0.004**	0.72 (0.65–0.79)	9.14	69	66
Preeclampsia	AIP	1.46 (1.08–1.99)	**0.013**	0.67 (0.60–0.74)	0.20	65	63
CAPO	TyG	1.78 (1.35–2.33)	**< 0.001**	0.80 (0.74–0.86)	9.16	75	71

Values are presented as adjusted odds ratios (aOR) with 95% confidence intervals (CI). Models were adjusted for maternal age, BMI, parity, smoking status, HbA1c, and fasting glucose. P-values < 0.05 were considered statistically significant. The discriminative performance of each index was assessed by ROC analysis, with area under the curve (AUC) provided

*AIP *atherogenic index of plasma, *TyG *triglyceride–glucose index, *FGR *fetal growth restriction, *NICU *neonatal intensive care unit, *CAPO* composite adverse perinatal outcome, *aOR *adjusted odds ratio, *CI *confidence interval

AIP showed an independent inverse association with FGR (OR 0.68, 95% CI 0.51–0.92, *p* = 0.013) but was not predictive of other adverse outcomes after adjustment.

### ROC curve analysis

**Fig. 3:**
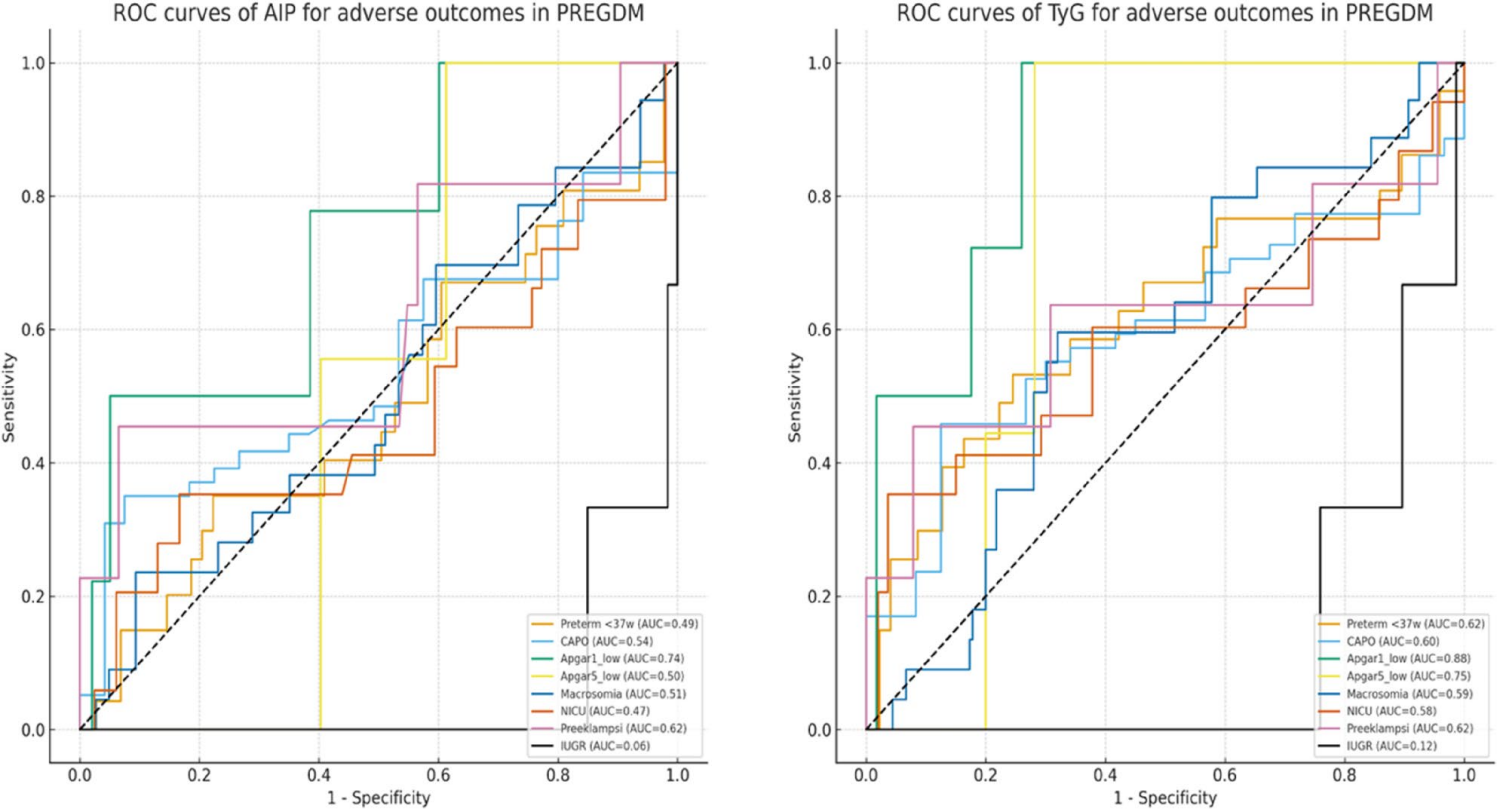
ROC curves of AIP and TyG for adverse outcomes in PREGDM

ROC curves are depicted in Fig. 3. The TyG index demonstrated the strongest discriminative ability for neonatal compromise. It predicted low Apgar at 1 min with an AUC of 0.88 and Apgar at 5 min with an AUC of 0.75, indicating good predictive performance. For preterm birth and NICU admission, AUCs ranged from 0.72 to 0.76.

AIP showed moderate predictive capacity for Apgar 1 min (AUC 0.74) and preeclampsia (AUC 0.67), whereas predictive performance was limited for CAPO and macrosomia (AUC 0.54–0.59).

Neither TyG nor AIP reliably predicted FGR (AUC < 0.60). Although AIP showed an independent inverse association with FGR in regression analyses, ROC analysis demonstrated poor discriminative performance, limiting its predictive utility.

**Fig. 4:**
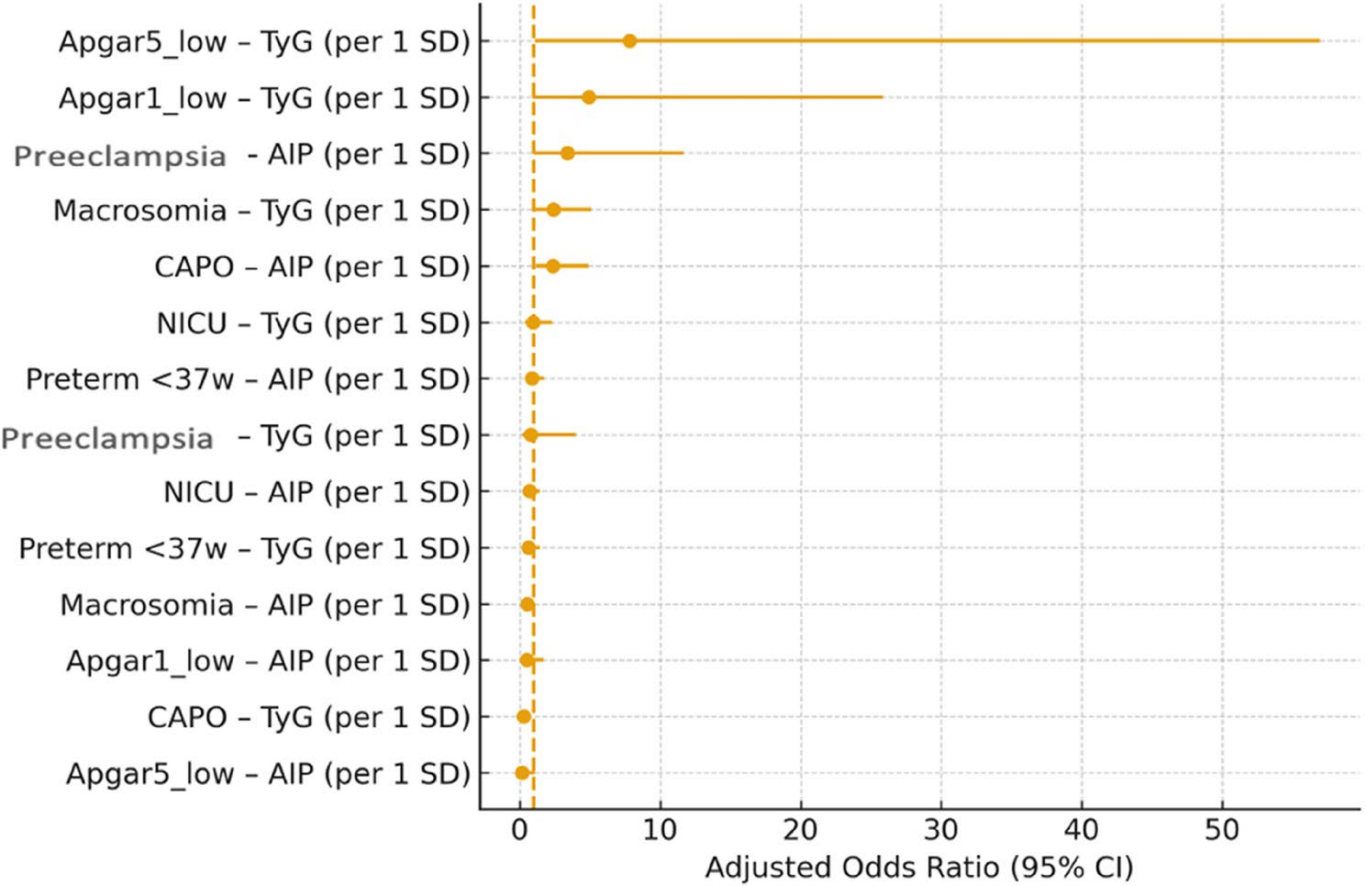
A summary of adjusted odds ratios per 1-SD increase in AIP and TyG across all adverse outcomes

A summary of adjusted odds ratios per 1-SD increase in AIP and TyG across all adverse outcomes is illustrated in Fig. 4.

A comparative summary of univariate and multivariate results is provided in Supplementary Table 1, illustrating which associations lost significance after adjustment.

## Discussion

In this study, both the Atherogenic Index of Plasma (AIP) and the Triglyceride–Glucose (TyG) index were significantly elevated in women with pregestational diabetes mellitus (PREGDM) compared with controls. TyG emerged as a strong independent predictor of preterm birth, macrosomia, low Apgar scores, and composite adverse perinatal outcome (CAPO), while AIP showed an inverse association with FGR and a positive association with low Apgar scores at 1 min. ROC analyses demonstrated particularly high discriminative power of TyG for neonatal compromise (AUC 0.88 for Apgar < 7 at 1 min). These findings emphasize that although both indices reflect metabolic alterations, they capture different aspects of risk in PREGDM pregnancies: TyG as a marker of insulin resistance–related metabolic stress and AIP as an indicator of dyslipidemia-driven vascular dysfunction. Importantly, TyG retained its independent predictive associations even after adjustment for HbA1c and fasting glucose, underscoring its incremental value beyond conventional measures of glycemic control.

### TyG index and adverse pregnancy outcomes

Our findings align with a large body of evidence supporting TyG as a powerful predictor of gestational and obstetric complications [25, 26]. Large population-based studies have demonstrated that first-trimester TyG is associated with a threefold increase in GDM risk, with an optimal cut-off around 8.5 (AUC ≈ 0.65) [27]. Other research has shown that TyG values above 8.6 are linked not only to GDM (aRR ≈ 1.9) but also to preeclampsia (aRR ≈ 2.2) [28]. Elevated TyG has also been confirmed as an independent risk factor for hypertensive disorders of pregnancy, low birthweight, and fetal distress [29, 30].

Li et al., in a study of 41,694 pregnancies, found that women in the highest TyG quartile had a 31% higher risk of preeclampsia and an 18% higher risk of preterm birth. While TyG alone had moderate predictive ability (AUC 0.596), its accuracy improved significantly when combined with blood pressure (AUC 0.736) [31]. Our results are consistent with this literature, particularly emphasizing TyG’s role in predicting preterm birth and neonatal adaptation failure.

Mechanistic investigations have provided further insight by demonstrating that the relationship between TyG and low birthweight is largely mediated through preterm delivery, which accounts for nearly 90% of the association [26]. This is directly in line with our observation that TyG predicts both preterm delivery and neonatal compromise. Lin et al. confirmed that elevated TyG independently doubled the risk of large-for-gestational-age (LGA) infants [32]. Additional studies have also reported that TyG thresholds above 4.88 and TG/HDL ratios above 3.63 are strong predictors of macrosomia, with excellent discriminatory performance (AUC ≈ 0.93) [33]. In our cohort, TyG was independently associated with macrosomia, although the discriminative accuracy for this outcome was modest, consistent with heterogeneity across studies.

Although the present study focused on women with PREGDM, in whom the diagnosis is established before conception, the early measurement of TyG in the first trimester may still provide valuable insight into baseline insulin resistance and metabolic stress that predispose to later complications. This supports the potential use of TyG as an early metabolic risk marker applicable even before overt hyperglycemia becomes clinically evident.

### The role of maternal BMI and novel indices

The predictive value of TyG is strongly influenced by maternal BMI. Evidence from very large cohorts has shown that overweight and obese women with elevated TyG face markedly increased risks of GDM, hypertensive disorders of pregnancy, large-for-gestational-age neonates, and preterm birth [31]. Longitudinal analyses have further demonstrated that persistently high TyG-BMI trajectories are powerful predictors of GDM (aOR ≈ 2.0), hypertensive disorders of pregnancy (aOR ≈ 6.0), and LGA (aOR ≈ 2.8), with stronger predictive performance than TyG alone (AUC ≈ 0.73 vs. 0.60) [25].

Validation studies in Asian populations have also confirmed the diagnostic utility of the TyG-BMI index, reporting excellent accuracy for GDM (AUC ≈ 0.80; cut-off ≈ 211; specificity 86%, sensitivity 67%) [18]. More recently, novel indices such as the TyHGB (TyG + HDL + BMI) have been proposed, demonstrating superior predictive accuracy for GDM compared with TyG alone (AUC ≈ 0.71 vs. 0.67 in Chinese cohorts; 0.86 vs. 0.81 in Korean cohorts) [34]. Collectively, these findings underscore that TyG-based composite indices, particularly TyG-BMI and TyHGB, may outperform TyG alone in early pregnancy screening and that maternal adiposity substantially amplifies TyG’s predictive impact.

### AIP, lipid abnormalities, and pregnancy outcomes

Pregnancy is characterized by physiological hyperlipidemia, which is accentuated in GDM and PREGDM. Cibickova et al. described pronounced elevations in triglycerides, low HDL, and small dense LDL particles in GDM, which directly contribute to macrosomia [16]. Our finding that higher AIP was inversely related to FGR may reflect this duality of maternal lipids: excess driving macrosomia and deficiency leading to restricted growth. This inverse association suggests that higher maternal lipid availability may favor fetal growth in PREGDM pregnancies, where relative lipid deficiency could limit substrate supply to the fetus despite maternal hyperglycemia.

Zhang et al. confirmed that AIP is positively associated with GDM risk in a Korean cohort, with strong predictive performance (AUC = 0.79; cut-off = 0.3557) [10]. This suggests that AIP may also serve as a metabolic risk biomarker, not limited to vascular complications. Agu et al. reported that AIP, CRR, and AC were significantly elevated in preeclampsia, with AIP >0.1 conferring an eightfold increased risk [15]. Yang et al. confirmed via systematic review that preeclampsia is consistently associated with low HDL and high VLDL, both of which are reflected in AIP [3]. Together, these findings position AIP as a marker of vascular dysfunction and hypertensive complications in pregnancy.

In our cohort, systolic and diastolic blood pressures were modestly but significantly higher in women with PREGDM compared with controls, despite all measurements being within the normotensive range. These early differences, derived from first antenatal visit records, may reflect subtle vascular dysfunction preceding overt hypertensive disease and are consistent with AIP’s role as a marker of endothelial stress.

Notably, a higher proportion of women with PREGDM exhibited proteinuria at baseline compared with controls, its presence may indicate early glomerular endothelial dysfunction or subclinical diabetic nephropathy. This finding is consistent with the concept that chronic vascular and renal microangiopathy may coexist in PREGDM pregnancies, contributing to altered hemodynamic regulation and the observed increase in AIP and blood pressure values.

### Beyond pregnancy: long-term implications of TyG and AIP

Beyond immediate perinatal outcomes, TyG and AIP have implications for long-term metabolic and reproductive health. Zou et al. showed that cumulative AIP exposure in prediabetic adults increased progression to overt diabetes and reduced reversion to normoglycemia [11]. Zhang et al. [7] and Karimpour Reyhan et al. [9] demonstrated associations between AIP and diabetic kidney disease and obesity. Bao et al. identified a strong positive association between AIP and infertility risk in women, with the highest quartile carrying a 2.38-fold increased risk [14]. These findings suggest that AIP captures systemic metabolic and vascular dysregulation with implications for both fertility and long-term health.

Similarly, Feng et al. reported that TyG was associated with metabolic syndrome, NAFLD, and reduced conception and live birth rates in PCOS [17]. This highlights TyG’s broader role as a systemic reproductive and metabolic risk marker, extending its relevance beyond pregnancy.

### Pathophysiological considerations

The divergence between TyG and AIP reflects distinct yet complementary biological pathways. TyG captures insulin resistance, hyperglycemia, and hypertriglyceridemia, leading to endothelial dysfunction, placental lipotoxicity, oxidative stress, and inflammation. This explains its strong associations with preterm birth, macrosomia, and neonatal compromise [35]. AIP, reflecting TG/HDL balance, is more closely linked to dyslipidemia-driven vascular stress, consistent with its associations with FGR and preeclampsia [36].

Maternal vascular malperfusion has been recognized as a key pathological substrate underlying preeclampsia, intrauterine growth restriction, and preterm birth, and it also serves as a predictor of maternal cardiovascular disease in the decade following pregnancy [5]. In this context, TyG and AIP may represent inexpensive biochemical markers of placental maladaptation, providing a practical link between adverse perinatal outcomes and long-term maternal cardiovascular risk.

### Clinical and translational implications

Both TyG and AIP are inexpensive, reproducible, and derived from routine laboratory tests. TyG correlates with HbA1c but predicted adverse outcomes independently of HbA1c and fasting glucose in our cohort, underscoring its incremental prognostic value. Prior studies also confirm that TyG provides predictive information beyond traditional glycemic markers, highlighting its role as a complementary biomarker [12, 20, 21]. AIP, on the other hand, is particularly useful in identifying vascular phenotypes prone to preeclampsia and growth restriction [3, 15].

Emerging evidence highlights the value of integrated risk models. TyG’s predictive accuracy improves when combined with BMI, blood pressure, or HDL, while longitudinal monitoring of TyG-BMI trajectories provides dynamic risk stratification [33, 34]. Such models could be incorporated into antenatal screening programs, offering early and cost-effective identification of high-risk women. Although logistic regression was selected for interpretability and comparability with prior obstetric literature, future studies could employ tree-based or machine-learning approaches (e.g., random forests, gradient boosting) to enhance discrimination and calibration. Combining AIP and TyG with hemodynamic or inflammatory markers may further improve clinical utility.

Beyond pregnancy, both indices may identify women at risk of infertility, diabetes, and cardiovascular disease, reinforcing pregnancy as a “stress test” for long-term health.

### Strengths and limitations

Our study benefits from a relatively large and carefully characterized PREGDM and control cohort, robust multivariate regression analyses, and ROC evaluation of predictive performance. However, limitations include the retrospective design, single-center population, and reduced power for rare outcomes such as stillbirth. In addition, information regarding insulin regimen, dosage, and use of lipid-lowering therapy (such as statins or omega-3 supplements) was not available, which may have influenced metabolic parameters and lipid indices.

Although the PREGDM group included both T1DM and T2DM, subgroup analyses by diabetes type were not performed due to limited sample size per subtype. Both entities share overlapping pathophysiologic mechanisms—including insulin resistance, oxidative stress, and endothelial dysfunction—that underlie adverse pregnancy outcomes; therefore, their combined analysis was considered appropriate for this initial investigation. Future multicenter studies with larger and more balanced cohorts will be essential to allow stratified analyses by diabetes type and to validate these preliminary findings.

## Conclusion

Our findings highlight that TyG is a robust independent predictor of adverse outcomes in PREGDM—including preterm birth, macrosomia, CAPO, and neonatal compromise—while AIP provides complementary insights into vascular dysfunction, FGR, and preeclampsia risk. Integrated with a broad literature base, these results suggest that TyG and AIP, along with novel derivatives such as TyG-BMI and TyHGB, should be considered in obstetric risk stratification. Future prospective multicenter studies are warranted to establish validated cut-offs, refine composite indices, and evaluate whether interventions targeting insulin resistance and dyslipidemia can improve both perinatal and long-term maternal outcomes. 

## Supplementary Information

Supplementary Material 1.

## Data Availability

The datasets used and/or analyzed during the current study are available from the corresponding author upon reasonable request.
