# Supplementary material for: First-trimester atherogenic index of plasma and triglyceride–glucose indices in pregestational diabetes mellitus: associations with adverse pregnancy outcomes
Source: BMC Pregnancy Childbirth. 2025 Nov 22;25:1351. doi: 10.1186/s12884-025-08491-2 (PMC12750775; doi:10.1186/s12884-025-08491-2)
Supplement: Supplementary file 1 — Supplementary Material 1. [file 12884_2025_8491_MOESM1_ESM.docx]

## **Supplementary Table: Comparison of Univariate and Multivariate Associations**

| Outcome | Index | Univariate (p<0.05) | Multivariate (adjusted) |
| --- | --- | --- | --- |
| Preterm birth | TyG | ✓ | ✓ |
| Macrosomia | TyG | ✓ | ✓ |
| CAPO | TyG | ✓ | ✓ |
| Low Apgar (1–5 min) | TyG | ✓ | ✓ |
| NICU admission | TyG | ✓ | borderline |
| Preeclampsia | AIP | ✓ | ✓ |
| FGR | AIP | ✓ (inverse) | ✓ (inverse) |
| CAPO | AIP | ✓ | ✗ (NS) |

This supplementary table summarizes the associations between AIP/TyG indices and adverse perinatal outcomes that were significant in univariate analyses (Table 3) and their corresponding multivariate results (Table 4). ✓ indicates significance at p<0.05; ✗ denotes non-significance after adjustment for confounders (maternal age, BMI, parity, smoking status, HbA1c, fasting glucose). NS: not significant. “Borderline” refers to p values between 0.05 and 0.10.
